# Niños genios, sabios venerables: el científico histórico en la literatura infantil y juvenil colombiana

**DOI:** 10.1590/S0104-59702026000100010

**Published:** 2026-06-01

**Authors:** Jorge M Escobar Ortiz

**Affiliations:** i Docente Ocasional de Tiempo Completo, Instituto Tecnológico Metropolitano. Medellín – Antioquia – Colombia. jorgeescobar@itm.edu.co

**Keywords:** Literatura infantil y juvenil, Científico histórico, Historia de la ciencia, Vocaciones científicas, Brechas de género, Children’s and young adult literature, Historical scientist, History of science, Scientific vocations, Gender gaps

## Abstract

La literatura infantil y juvenil suele concebirse como una estrategia para iniciar a los niños y los jóvenes en los diálogos culturales sobre la ciencia. Esto redundaría en la promoción de las vocaciones científicas y la reducción de las brechas de género. Este artículo muestra cómo la tradición literaria colombiana ha promovido dichas vocaciones mediante una mezcla de instrucción moral y entretenimiento racional que representa al científico histórico como un sabio, un genio y un niño autodidacta incomprendido por su sociedad. Surge la cuestión de la conveniencia de este imaginario para abordar las vocaciones científicas y las brechas de género.

## Abstract (en)

Children’s and young adult literature is often seen as a means to introduce this public to cultural discussions about science. This would aim to promote scientific careers and reduce gender gaps. The article shows how Colombian literary tradition has facilitated this goal by combining moral instruction and rational entertainment, portraying the historical scientist as a wise, gifted, and self-taught child misunderstood by society. This raises the question of whether this imagery addresses scientific careers and gender gaps.

La ciencia para niños y jóvenes es un campo que ha ganado relevancia en los estudios de ciencia y tecnología, particularmente por su importancia para comprender los procesos de popularización científica en diferentes grupos y contextos nacionales. Se ha analizado su lugar en los programas de televisión (Carvalho et al., 2020), los cómics y el cine (Silva Filho, Massarani, Stengler, 2021; Silva Filho, Massarani, 2024), los periódicos (Zilhão, 2014), las revistas, las enciclopedias y otra variedad de medios y formatos (Zilhão, Nielsen, 2018). La literatura infantil y juvenil (LIJ) es una de sus expresiones más representativas.

A mediados del siglo XVIII, el editor británico John Newberry publicó una serie de libros sobre Tom Telescope, un niño que explicaba los conceptos de la física newtoniana a sus amigos en el Londres del momento. Estos libros, que tuvieron múltiples ediciones en varios idiomas hasta la primera mitad del siglo XIX, se consideran el origen moderno de la LIJ. Y como sostiene Debbie Bark (2017, p.187), “aquello particularmente interesante de esta narrativa de la historia de la literatura infantil y juvenil es que desde su concepción está intrínsecamente conectada con la ciencia para niños”.^1^ En un artículo pionero y bastante influyente en este ámbito, James A. Secord (1985, p.128) sostuvo que

los libros de literatura infantil y juvenil merecen un lugar importante en la historia de la ciencia. Interpretados cuidadosamente, proporcionan indicadores invaluables de los cambiantes valores sociales, religiosos y morales que lleva el conocimiento científico en diferentes circunstancias. Tal como ahora los historiadores rutinariamente usan los libros de texto para trazar los contornos del consenso contemporáneo en una comunidad científica, así los libros de literatura infantil y juvenil pueden emplearse a un nivel más general para retratar el rostro aceptable de la ciencia como lo ven grupos sociales mucho más amplios entre el público que compra libros.

Secord (1985, p.139) se concentró en el origen y la recepción de los libros de Tom Telescope y encontró que ayudaron a que la ciencia entrara en los círculos familiares mediante una “mezcla de instrucción moral y entretenimiento racional”.

Esta mezcla de instrucción moral y entretenimiento racional caracteriza a la LIJ en su desarrollo posterior, al entenderse como un género que incorpora valores educativos. Clarice Fortkamp Caldin (2003, p.50) resalta su “función formadora”, al presentar “modelos de comportamiento que facilitan la integración del niño en la sociedad”. Eliana Lucía Sepúlveda Montenegro (2021, p.52) señala su vínculo con “la necesidad de transmitir la herencia cultural, social y económica de la sociedad en la que se inscribe”. Y Rocío Vélez de Piedrahita (2021, p.26) apunta que “toda obra, aunque no tenga por fin enseñar, enseña las palabras que utiliza, las ideas que expone, los sentimientos que presenta; por lo tanto, la poesía, los cuentos y aventuras de calidad son tremendamente útiles para la formación y desarrollo de los niños”. Para Teresa Colomer (2010, p.15), la LIJ supone la iniciación de los niños y los jóvenes al diálogo cultural de su sociedad y esto se logra por ciertas funciones del género:

Aquí se sostiene que la literatura para niños y jóvenes debe ser, y verse, como literatura, y que las principales funciones de estos textos pueden resumirse en tres:

1. Iniciar el acceso al imaginario compartido por una sociedad determinada.

2. Desarrollar el dominio del lenguaje a través de las formas narrativas, poéticas y dramáticas del discurso literario.

3. Ofrecer una representación articulada del mundo que sirve como instrumento de socialización de las nuevas generaciones.

La relación con la ciencia destaca, en definitiva, no solo en los orígenes del género, sino en la definición de su intención educativa. La LIJ aparece como una estrategia para aculturar a los niños y los jóvenes en su propia sociedad, con la ciencia – término que se emplea aquí para denotar el conjunto que conforma con la tecnología – como un componente importante de dicha sociedad. Aun así, esta relación entre la LIJ y la ciencia ha recibido poca atención en español. El mayor énfasis puede identificarse en trabajos que, a partir del comentario de algunas obras puntuales, no siempre escritas en esta lengua, valoran la LIJ como una estrategia intrínsecamente positiva para promover las vocaciones científicas en niños, niñas y jóvenes, lo que además redundaría en la disminución de las brechas de género en la ciencia (Figueiras, Esteve, 2009; De Sarlo, 2017; Calvo Iglesias, 2019; Guevara Ruiseñor, 2023).

Este ensayo no niega el potencial del género para tales propósitos, pero complejiza su mirada con un acercamiento más global a una tradición literaria. El caso de la LIJ colombiana permite indagar cómo esta tradición habría promovido las vocaciones científicas desde finales del siglo XX mediante el uso de ciertos imaginarios sobre el científico histórico, es decir, aquellas personas que vivieron en algún momento del pasado y hacen parte de la historia de la ciencia nacional o internacional. El científico histórico se entiende en contraposición al científico ficticio, aquellos científicos creados como personajes de ficción que aparecen en otras obras del género. Roslynn D. Haynes (1994, 2003) argumenta que la literatura occidental ha presentado al científico ficticio según seis o siete estereotipos recurrentes que enfatizan una mirada negativa de los científicos y la ciencia en general. El análisis de la LIJ colombiana sugiere una perspectiva opuesta: una mirada bastante positiva del científico histórico que lo representa incluso como un modelo intelectual y ético para la sociedad. Así, una pregunta soporta este acercamiento: ¿qué imaginarios del científico histórico presenta la LIJ colombiana a sus lectores? Lectores que son niños y jóvenes, pero también adultos – escritores, padres, docentes, bibliotecarios, promotores de lectura, por mencionar algunos – que eligen unos libros y no otros, que los compran o los prestan para leerlos y comentarlos con esos niños y esos jóvenes, como parte del proceso de aculturación mencionado antes. Es, en últimas, una pregunta por cómo el género incide en la formación y la consolidación de ciertos imaginarios científicos en la sociedad.

Esta mirada a la LIJ colombiana se justifica porque, a pesar de ocupar, como el resto de la LIJ latinoamericana, un lugar periférico en el contexto literario internacional (Guijarro Arribas, 2021), es una tradición que tampoco ha sido ajena a la ciencia. Esto puede ser de interés para el acercamiento a tradiciones semejantes en lengua española, particularmente en América Latina. En Colombia, los estudios de teoría literaria han investigado el concepto de LIJ y su inclusión en el canon nacional (Borja Orozco, Alonso Galeano, Ferrer Franco, 2010; Borja Orozco, Alonso Galeano, 2015, 2018), mientras que los estudios históricos han ofrecido panoramas generales desde el siglo XIX hasta la primera década del siglo XXI (Díaz, 1999; Pardo Chacón, 2009; Robledo, 2012). En todos los casos, la ciencia se ha excluido de los temas de interés en el país.

Esto es llamativo porque existe evidencia de que la ciencia ocupa un lugar central en la LIJ colombiana, tanto en su vertiente de obras informativas (no ficción) como en aquella de obras literarias (ficción) (Escobar Ortiz, 2023). Incluso desde el siglo XIX y durante el siglo XX, como se infiere de varios trabajos (Robledo, 2004, 2012; Aristizábal García, 2018; Peters, Trujillo Acosta, 2018; Cifuentes Álvarez et al., 2021; Sepúlveda Montenegro, 2021), es posible rastrear obras de temas científicos en publicaciones periódicas que dedicaban algunas secciones a la LIJ, como *La Caridad* (1864-1914, con interrupciones) o *La Mujer* (1878-1881), o se dedicaban exclusivamente a ella, como *El Álbum de los Niños* (1871-1893, con interrupciones), *La Niñez* (1914-1917), *Chanchito* (1933-1934) y *Rin Rin* (1935-1939). Dada la falta de un trabajo sistemático de archivo sobre dichas publicaciones, aquí se toma como referencia un conjunto de obras publicadas desde la segunda mitad del siglo XX en el formato de libro. El listado completo, que se apoya en un corpus previo (Escobar Ortiz, 2023), se presenta en las Tablas 1 y 2.

Este conjunto de obras permite concluir que, en la LIJ colombiana contemporánea, existe una fuerte inclinación a representar al científico histórico no según los estereotipos negativos del científico ficticio (Haynes, 1994, 2003), sino según un estereotipo positivo que lo proyecta como un sabio, un genio y un niño autodidacta que tiende a ser incomprendido por su medio social, aunque esto no implique una contraposición fundamental con dicho medio. La promoción de las vocaciones científicas y su influencia en fenómenos como la reducción de las brechas de género se enmarcan en dicha representación. Las obras apelan a figuras prominentes de la ciencia nacional y mundial para popularizar un imaginario del científico en línea con esa mezcla de instrucción moral y entretenimiento racional que señala Secord para un científico ficticio como Tom Telescope. Los científicos históricos se convierten en ejemplos que los lectores pueden y deben emular no solo por el valor de sus intereses intelectuales y sus descubrimientos, sino especialmente por su rectitud ética hacia la ciencia, sus congéneres e incluso otros seres vivos. Esta mirada resalta muy pronto en la Serie Juvenil de Colciencias.

Aparecida entre 1995 y 2003, es el proyecto editorial de LIJ de temas científicos más importante del país. La publicó el entonces Instituto Colombiano para el Desarrollo de la Ciencia y la Tecnología “Francisco José de Caldas” (Colciencias), antecedente del actual Ministerio de Ciencia, Tecnología e Innovación (Minciencias), y convocó a escritores e ilustradores colombianos de gran prestigio. La serie estuvo concebida y dirigida por Carlos Nicolás Hernández Camacho, que también estaría a cargo de la Biblioteca Colombiana Ilustrada, un proyecto de orientación semejante. El resultado fueron las biografías noveladas/novelas biográficas de 21 figuras prominentes en la historia de la ciencia colombiana, todas escritas bajo un presupuesto común. Así lo describe Patricia Aguirre (2004, p.84-85), una de las encargadas de coordinar dicha serie:

Los relatos de cada volumen de la serie juvenil de Colciencias buscan ir más allá de su obra científica, buscan conocer la vida del personaje, vida ejemplar que se convierte en un elemento dinamizador de los procesos de cambio social, del desarrollo de una cultura política participativa, a la vez que se convierte en una manera de incentivar en los jóvenes lectores el amor por la ciencia.

La serie destaca inmediatamente por los nombres de quienes firman los textos. Junto a autores mayores del género en el país, como Jairo Aníbal Niño, Gonzalo España, Celso Román, Pilar Lozano, Beatriz Caballero, Irene Vasco y Yolanda Reyes, se unieron otros como Germán Espinosa, Sandro Romero y Antonio Orlando Rodríguez y académicos como Santiago Díaz Piedrahita, Carlos Andrés Barragán y Carolina Alzate. Así que, aunque comparten algunos horizontes estéticos y temáticos, se notan diferencias en énfasis y calidad literaria, que no rompen la unidad de la colección. Cada biografía novelada emplea recursos estilísticos propios. En algunas prevalece una tercera persona omnisciente, en otras una tercera persona que acompaña a los personajes sin saberlo todo de ellos. Otras dejan hablar a los personajes desde la primera persona, de viva voz o desde sus cartas, diarios y otros documentos. Hay viajes en el tiempo y objetos que narran la vida de los científicos. Cabe mencionar la calidad gráfica de los libros, su diseño y sus ilustraciones. Las ilustraciones cubren la página, abrazan el texto y dialogan con él, recurriendo a imágenes de archivo y reproducciones de pinturas de cada época, con la pretensión de dar un sentido de realidad histórica a aquello que narra el texto. Un intento por mostrar el pasado y el conocimiento científico de manera objetiva, tal como sucedieron.

También destaca el período cubierto desde poco antes de la Independencia hasta finales del siglo XX. Los científicos colombianos no se reducen a José Celestino Mutis y su círculo de la Expedición Botánica, como suelen enfatizar la historiografía y la literatura nacionales. Se incluyen figuras posteriores, hombres y mujeres, pertenecientes a las ciencias naturales y las ingenierías, incluso las ciencias sociales y humanas, sin que se perciban tensiones. Aquí cabe señalar la presencia de científicos extranjeros, todos visitantes del país en algún periodo de su historia. Sin contar las novelas biográficas sobre José Celestino Mutis y Agustín Codazzi, que tienden a asumirse como colombianos, se encuentran otras sobre Alexander von Humboldt (España, 1996), Claude Véricel (Román, 1997), Francisco Javier Cisneros (Lozano, 1997a) y Jean Baptiste Boussingault (España, 1998c), entre otros. Estas obras comparten las características generales de la colección, aunque al ocuparse de científicos extranjeros que entran y salen de países y territorios, logran representar su trabajo en contextos supranacionales que contribuyen a ver la ciencia y la circulación del conocimiento desde perspectivas que pueden describirse como trasnacionales.

La serie tampoco rehúye los contextos culturales, económicos y políticos que rodean al científico. Esto es importante porque frente a los intentos teóricos y prácticos por defender la neutralidad de la ciencia, aislarla de esos contextos y desacreditar la labor científica que “se politiza”, las obras insisten en mostrar los conflictos, los desencuentros, las luchas de poder y su relación con la producción científica. El científico no es un ser aislado, dedicado a pensar y resolver los problemas de la humanidad sin relacionarse con ella. Es un ser político, sin que esto implique una disminución intrínseca de su labor. Las guerras nacionales y continentales de América y Europa enmarcan obras como *Paul Rivet: estudioso del hombre americano*, de Antonio Orlando Rodríguez (1998), y *José Jerónimo Triana: el caballero de las flores*, de Santiago Díaz Piedrahita (1999). Esto obliga al científico a tomar partido, no se le permite ser indiferente, incluso se vincula a algunos gobiernos. Sus inclinaciones políticas no lo hacen menos científico. Antes parecen asumirse como una extensión de su trabajo.

El científico también se trata como un ser humano con algunas debilidades personales. Puede gustarle la bebida un poco de más, ser marrullero o despistado, buen o mal negociante. Nada de eso nubla su grandeza. De hecho, todas las obras se detienen en ese punto, tímidas o temerosas de intentar algún avance ulterior. El científico es en todos los casos, más allá de cualquier tara menor, un sabio cuyo brillo ilumina a sus congéneres. Tiene la cualidad de no desfallecer ante las adversidades y triunfar en las empresas que emprende, además de presentarse como una especie de faro moral que, contra las resistencias de su entorno, logra imponer la versión correcta de los fenómenos, sin motivaciones personales o egoístas, por el bien de la humanidad. Esto es notorio en los dos libros de Germán Espinosa. En *Lino de Pombo: el sabio de las siete esferas*, Pombo fue capaz de oponerse a las tendencias dictatoriales de Bolívar porque “mi verdadera vocación no son las armas, sino las matemáticas. Estoy seguro de poder servir mejor a la patria en una cátedra” (Espinosa, 1998a, p.74), mientras que en *Federico Lleras Acosta: la guerra contra lo invisible*, se insiste en el desinterés de Lleras Acosta por la fama y el dinero, pues solo lo movía el beneficio de sus investigaciones médicas para los demás (Espinosa, 1998b, p.9).

Pero el científico es ante todo un niño científico. Se repite sin cesar, compulsivamente, que su genialidad, su inteligencia superior, se manifiesta en la niñez. En *José María Villa: el violinista de los puentes colgantes*, de Pilar Lozano (1997b, p.61), se asegura que Villa, “desde pequeño, había dejado muy en claro que el tamaño de su curiosidad era gigante y que para él nada estaba prohibido por más riguroso que fuera”. Uricoechea exhibe desde niño una sensibilidad superior, “una curiosidad insaciable” y una gran inteligencia, como se ve en *Ezequiel Uricoechea: el niño que quería saberlo todo*, de Celso Román (1998, p.10, 11, 28). En *Alejandro López: a la medida de lo imposible*, de Irene Vasco (1998, p.10-14), López muestra, desde niño, a diferencia de sus hermanos, placer y curiosidad por las matemáticas, algo semejante a Garavito, que es un genio matemático y científico desde la niñez, como lo describe *Julio Garavito: de Colombia a la Luna*, de Sandro Romero (1998, p.9, 33-34). Y Uribe Ángel, antes de los 10 años, despliega un gran gusto por el estudio de la naturaleza y pronto por la medicina, de acuerdo con *Manuel Uribe Ángel: el médico y geógrafo que amó a su país*, de Pilar Lozano (1998, p.6, 7-12). Ninguno de los científicos de la colección se representa como un niño o una niña con ocupaciones o intereses mundanos. Son pequeños sabios que dedican su tiempo a los campos de estudio por los que luego ganarán reconocimientos, una suerte de preformismo donde aquello que se admira del adulto ya estaba contenido en el infante.

La Serie Juvenil de Colciencias ofrece, en conjunto, una versión múltiple y vasta, aunque un tanto unilateral, del científico. No lo separa de su medio, no demoniza sus vínculos con la política y otros factores contextuales. Pero sí tiende a enfatizar algunos lugares comunes que privilegian el imaginario del hombre blanco, sabio, virtuoso y abnegado, en la tradición del sabio Mutis y el sabio Caldas, y de esa ciencia triunfal e impoluta que ha agradado al órgano rector de la política científica en el país, llámese Colciencias o Minciencias. La figura del científico histórico es, ante todo, un ejemplo para las nuevas generaciones, un objeto de culto y veneración cuya efigie debe admirarse y que pretende representarse de manera fidedigna y objetiva, incluso con las ilustraciones. Estos elementos, que ostentan esa mezcla de instrucción moral y entretenimiento racional que ya se ha comentado, resaltan aún más al considerar los dos proyectos científicos más influyentes de la historia del país y sus tres científicos prominentes: José Celestino Mutis, Francisco José de Caldas y Agustín Codazzi.

Los dos primeros aparecen como las figuras emblemáticas de la Expedición Botánica al Nuevo Reino de Granada (c.1783-1816), el proyecto científico que se describe con frecuencia como el motor de la Independencia y el surgimiento de la nación. La Expedición Botánica reunió a un grupo de criollos ilustrados que trabajó de la mano con otros grupos pertenecientes a diferentes tradiciones locales, tanto criollas como campesinas, artesanales, negras, indígenas y mestizas, con y sin reconocimiento explícito de sus aportes (Puerta Olaya, 2025). José Celestino Mutis ocupó el puesto central como fundador y líder de la expedición, por sus esfuerzos para difundir el newtonianismo, el sistema linneano, la medicina europea y otros estandartes de la ciencia moderna. No sorprende que, con un buen número de obras, ocupe el lugar más destacado entre los científicos históricos en la LIJ colombiana de temas científicos.

Gonzalo España (1998a, 1999, 2008, 2017) ha contribuido bastante, con trabajos pertenecientes a diferentes géneros, como la biografía novelada *José Celestino Mutis: el sabio de la vacuna*, de la Serie Juvenil de Colciencias, la biografía *Mutis y la Expedición Botánica* y las novelas históricas *Los pies en la tierra, los ojos en el cielo* y *La última correría del sabio Mutis*. También Elisa Mújica (1978, 1985, 1993) exploró la figura de Mutis en su ensayo *La Expedición Botánica contada a los niños* y los cuentos “José Celestino y el dragón” y “Aventuras del príncipe Celestino”. A ellos se suman Beatriz Caballero (1983) con el ensayo *La aventura de la Expedición Botánica* y Celso Román (2010) con la biografía novelada *Mutis y la minería: amorosa mutisia*, de la Biblioteca Colombiana Ilustrada.

Todas estas obras concuerdan en su idealización de Mutis como un sabio con múltiples facetas, bondadoso, exitoso en sus empresas a pesar de las adversidades, faro moral y racional de la Nueva Granada, oráculo del Reino e incluso, aunque distante de los conflictos políticos, causa directa de la Independencia. Como asegura un personaje de *Los pies en la tierra, los ojos en el cielo,* de Gonzalo España (2008, p.113), Mutis “llegó aquí buscando la ciencia y la libertad, y ambas cosas nos dejó: la ciencia y la libertad”. Los dos cuentos de Elisa Mújica también hacen explícito el tema del niño sabio. En “José Celestino y el dragón”, por ejemplo, la sabiduría de Mutis se manifestó “desde chiquito” (Mújica, 1985, p.1).

Sin embargo, bajo esta reiteración de motivos ya familiares en el imaginario nacional, que evitan problematizar uno de sus mitos fundacionales, logran mezclarse algunas novedades que no carecen de interés. Un primer elemento descentra el foco de la Expedición Botánica, sin olvidarla como marco general, y considera otras vertientes de la obra de Mutis. En sus biografías noveladas, Gonzalo España (1998a) se vuelca sobre los procesos de vacunación en la Nueva Granada, mientras que Celso Román (2010), desde una perspectiva bastante imaginativa, que da voz a las hormigas tantas veces estudiadas por Mutis, se vuelca sobre la entomología y la minería. Otro elemento es el reconocimiento explícito de las tradiciones locales como fuente directa para la producción científica de Mutis y su círculo de la Expedición Botánica, aunque sin especificar a qué grupos concretos harían referencia dichas tradiciones, más allá de describirse como indígenas, negras o mestizas. Esas tradiciones tienen un lugar destacado en las obras de Elisa Mújica (1978, 1985, 1993) y Beatriz Caballero (1983), pero particularmente en la bella novela *La última correría del sabio Mutis*, de Gonzalo España (2017), que narra las investigaciones de los ilustrados neogranadinos sobre las lenguas indígenas y expone, mediante el personaje del indio Tancredo Rancio, la influencia de los conocimientos locales en Mutis y sus discípulos durante toda la Expedición Botánica. Por último, se insinúa, sin desarrollarlo a profundidad, el interés económico de la Corona española en los trabajos de Mutis, con lo que la Expedición Botánica no se entiende exclusivamente como un proyecto científico, sino también comercial, militar y político. El ensayo de Beatriz Caballero (1983) dedica algún espacio a este tema, mientras que los cuentos de Elisa Mújica (1985, 1993) lo abordan con la figura del dragón de la ignorancia, fuente de retraso para la Nueva Granada y némesis de Mutis.

Francisco José de Caldas, esa gran obsesión nacional, es el otro científico vinculado a la Expedición Botánica con un lugar sobresaliente en la LIJ colombiana. La Serie Juvenil de Colciencias incluye dos biografías noveladas: *Caldas: el inventor de lunas*, de Jairo Aníbal Niño (1995), que inaugura la colección y es quizá la mejor versión literaria que se ha escrito sobre él, incluidas las obras para adultos, y *Pierre Bouguer: el maestro del sabio*, de Gonzalo España (1998b). A estas se suman el ensayo *Caldas: precursor del patriotismo científico*, de Andrés Olivos Lombana (1998), la biografía *¡Soy Caldas! Biografía de Francisco José de Caldas*, de Stefan Pohl Valero (2009), y la elegante historieta *Francisco José de Caldas: un camino a las estrellas*, de Álvaro Vélez (truchafrita) y Gabriel Jaime Gómez Carder (2019).

Caldas se ha presentado tradicionalmente, en los trabajos históricos y en las obras literarias, mediante una dicotomía entre el precursor científico y el prócer político (Escobar Ortiz, 2022). Caldas, el precursor, se asume como el primer científico colombiano, a pesar de la existencia previa de la Expedición Botánica y a que Colombia no se había constituido como Estado nacional cuando murió. El primer colombiano en destacarse en múltiples campos científicos, todo realizado de manera autodidacta. Las obras reafirman este imaginario. Por ejemplo, es difícil percibir como casual el hecho de que la Serie Juvenil de Colciencias inicie con un volumen dedicado a Caldas. Por su parte, la historieta lo entroniza desde la introducción como el primero en todo: sus “actividades demostraron que el neogranadino es el primer científico de Colombia y precursor, entre nosotros, de disciplinas como astronomía, geografía de las plantas, ingeniería, hipsometría, meteorología y periodismo científico” (Vélez, Gómez Carder, 2019, p.2). Caldas fue el primer científico del país, el primero en el tiempo y el primero en grandeza.

En esa dicotomía que establecen la historiografía y la literatura, se muestra que, desafortunadamente, la labor del precursor se vio truncada por Caldas, el prócer, que se inmiscuyó en las turbaciones revolucionarias hasta hundirse en ellas y perecer. Esto explicaría la paradoja de que el primer científico colombiano, el precursor de la ciencia nacional, no haya completado ninguna empresa científica de peso significativo, más allá de los prospectos que sugieren sus cartas y sus obras, casi todas inéditas al morir. Sin embargo, la LIJ colombiana se aleja en este punto de una versión rígida de la dicotomía y brinda una versión más compleja del científico.

Las obras concuerdan en mostrarlo como un sabio cuyos intereses científicos estaban predefinidos en su niñez, al punto de que en *Pierre Bouguer: el maestro del sabio,* de Gonzalo España (1998b, p.5-6), el bebé Caldas, antes de aprender a caminar, evidenciaba una inclinación tan inusual hacia la observación de la naturaleza que, en lugar de meterse los insectos y los bichos a la boca cuando los cogía, se quedaba contemplándolos. Caldas se describe como un autodidacta que debe sobreponerse a la escasez mental y material del Virreinato, aun cuando se menciona su formación en los espacios educativos de la élite de su tiempo. Pero se rechaza la división tajante entre el precursor y el prócer. De ahí que, en *Caldas: precursor del patriotismo científico,* de Andrés Olivos Lombana, se sugiera desde el propio título que Caldas tuvo la capacidad de combinar con éxito sus facetas de científico y político: “En Caldas la investigación científica – y es su didáctica de mayor relevancia – se encuentra unida a un propósito patriótico y social: mejorar las condiciones de vida de los habitantes con base en el aprovechamiento de los recursos de la geografía” (Olivos Lombana, 1998, p.10). El Caldas político no invalida al Caldas científico, como también lo muestra *¡Soy Caldas! Biografía de Francisco José de Caldas,* de Stefan Pohl Valero (2009). Son aspectos complementarios que deben atenderse simultáneamente para comprender su importancia en la historia del país.

Tras la Expedición Botánica, el otro proyecto científico de gran envergadura e influencia en la historia de Colombia fue la Comisión Corográfica (c.1850-1859) (Sánchez, 1999; Appelbaum, 2016). La Comisión tuvo como foco la producción de conocimiento geográfico, cartográfico y corográfico, pero permitió también el desarrollo de conocimientos botánicos, antropológicos, sociológicos, ingenieriles, raciales, de género, políticos y económicos, además de obras literarias, plásticas y divulgativas. Su figura principal fue Agustín Codazzi, un ingeniero militar de origen italiano que participó en las guerras napoleónicas y las guerras bolivarianas. Tras asentarse en Venezuela, terminó por reconocerse como un cartógrafo de prestigio internacional gracias a su atlas de aquella nación. Con los cambios políticos vividos allí, aceptó liderar la Comisión Corográfica de la República de la Nueva Granada – uno de los nombres que recibió la actual Colombia en el siglo XIX – para crear un atlas mucho más ambicioso y preciso que el venezolano. Su muerte, en 1859, implicó el final del proyecto original, aunque obras geográficas y botánicas, apoyadas en los datos de la Comisión, se publicaron en las décadas siguientes.

La presencia de Codazzi en la LIJ colombiana se debe principalmente a Beatriz Caballero, que le dedicó el poema biográfico *Codazzi, el señor que dibujaba mapas* (Caballero, 1994a), la biografía *Las siete vidas de Agustín Codazzi* (Caballero, 1994b) y la biografía novelada *Codazzi: el siete leguas* (Caballero, 1997), incluida en la Serie Juvenil de Colciencias. A estas se suma la biografía novelada *Agustín Codazzi: el caballero andante*, de Carlos Nicolás Hernández Camacho (2010), parte de la Biblioteca Colombiana Ilustrada, una colección semejante a la Serie Juvenil de Colciencias, excepto que incluyó obras publicadas previamente y otras originales, como esta. Aunque es un volumen de un diseño editorial impecable, sus ilustraciones muestran también la pretensión de representar el pasado objetivamente, tal como fue, mediante imágenes y pinturas de la época, mientras que el texto resulta bastante dependiente de los trabajos de Beatriz Caballero.

En conjunto, estas obras evitan la idealización y presentan a Codazzi como un personaje complejo: militar, científico, político, aventurero, jugador, comerciante, padre de familia. Su presencia en las guerras napoleónicas y bolivarianas no choca con su labor como geógrafo e ingeniero. Ambas empresas se respaldan mutuamente. Forman un círculo virtuoso donde su papel de militar le permite trabajar como ingeniero y geógrafo y este papel de ingeniero y geógrafo le permite ascender como militar y ganar más peso y prestigio social. La importancia de su trabajo científico se resalta por la comparación con Cristóbal Colón y Alexander von Humboldt. Así lo hace Beatriz Caballero (1994b, p.26) en *Las siete vidas de Agustín Codazzi:* “Fue él quien verdaderamente descubrió el continente, más que Humboldt y que el mismo Colón – que no estuvo sino por la orillita y ni siquiera se enteró de que estaba en América”.

Tampoco aquí hay una oposición específica entre la ciencia y la política, aunque sí la tendencia, recurrente en otras obras, a entronar al científico como un modelo intelectual y moral para los lectores. *Codazzi: el siete leguas,* de Beatriz Caballero (1994a, p.49), menciona que a Codazzi no le importaban las incomodidades de sus recorridos con la Expedición, lo central era completar la labor científica. Además, destaca con fuerza la presencia de los hijos de Codazzi como ayudantes en sus tareas cartográficas y corográficas. Un elemento muy llamativo es el personaje de Constanza Codazzi, la niña que narra y escribe un diario sobre los trabajos y la vida de su padre. Es una niña curiosa, con muchos intereses en la ciencia, que incluso ayuda en la elaboración de los mapas. Constanza admira a su papá y transmite esa admiración al lector. Tiene el papel del niño/joven lector curioso que se acerca al libro y la vida del científico.

La mezcla de instrucción moral y entretenimiento racional caracteriza, en suma, la manera como la LIJ colombiana ha presentado estos dos proyectos científicos y sus personajes representativos. Igual sucede con la figura más notoria en la ciencia colombiana desde la segunda mitad del siglo XX. Manuel Elkin Patarroyo ha recibido respeto y reconocimiento por sus investigaciones en inmunología y patología, aunque su efigie manifiesta algunas grietas por cuestionamientos sobre el trato a los animales, sus formas de financiación y sus aportes al desarrollo de una vacuna contra la malaria, entre otras razones. La biografía novelada *El río de la vida: Manuel Elkin Patarroyo y su lucha contra la malaria*, de Jairo Aníbal Niño (1994), va en contravía de dichas grietas. Con las mismas tendencias idealizantes de otras obras, esta combina voces narrativas, siempre desde el punto de vista de los animales de la selva amazónica, que hablan del científico con cariño y admiración. El científico es un investigador incansable, un ser humano con una inteligencia superior, cuyo sueño por la ciencia inició en la niñez. Desde entonces, ha “jugado” a desarrollar la vacuna (p.34).

Patarroyo aparece como respetuoso y bondadoso con los animales, tanto que se ofrecen voluntariamente para que experimente con ellos, pues su sufrimiento queda justificado por el bien mayor: el bienestar de la humanidad. En su laboratorio no se establecen jerarquías, animales y humanos se unen por lazos de amistad, a diferencia de los factores contextuales – cazadores ilegales, problemas de financiación, escasez de recursos, incomprensiones y envidias – que se alzan en oposición a Patarroyo y este debe sortear: “¿No ven cómo este hombre conserva un temblor transparente en su alma? Él no sólo está luchando contra la malaria. Lo hace también contra el pesimismo, la pobreza, la mezquindad y el horror” (Niño, 1994, p.27). Cuando obtiene la vacuna, su primer acto es dirigirse a la selva a contarles y agradecerles a los animales, que lo reciben fulgurantes. En definitiva, más allá de sus virtudes literarias, la obra busca celebrar el nombre de Patarroyo y ampliar su rango de influencia en el imaginario científico del país, sin considerar ningún elemento que pueda salpicarlo con miradas críticas.

Otras figuras de alcances más globales han recibido tratos semejantes en la LIJ colombiana. Dos biografías tienen un carácter un tanto singular en este sentido. *Galileo Galilei, y sin embargo se mueve*, de German Puerta Restrepo (2005), y *Maquiavelo: ¿un científico moderno de la política?*, de Enrique Ferrer Corredor (2018), llaman la atención por el peso de sus personajes y porque no es usual encontrar figuras de la Modernidad temprana en la LIJ colombiana. Galileo se representa como un autodidacta que definió su inclinación por la ciencia desde niño. Es un genio que no actúa por vanidad, sino por el bien de sus congéneres. Su talento es tan grande que hasta sus dibujos de la Luna muestran que pudo ser artista si lo hubiera deseado. Su modernidad le hacía inevitable entrar en confrontaciones religiosas. Esto explica los ataques de sus contemporáneos, causados por envidia, celos e ignorancia. Aun así, la ciencia moderna le debe todo. Maquiavelo se describe de una manera semejante, incomprendido en su tiempo, caído en desgracia, pero reconocido por la posteridad como un genio del pensamiento político. De ahí su relevancia para que los jóvenes latinoamericanos se acerquen desde él a las problemáticas actuales. Cabe destacar que se tome a Maquiavelo como un antecedente de la ciencia moderna, no por el lado de las ciencias naturales como Galileo, sino de las ciencias sociales. En todo caso, ambas obras se plantan como impulsoras, más que críticas, de estas figuras entre los lectores.

Otras obras de carácter informativo, ya no ubicadas en los albores de la Modernidad, sino en los siglos XX y XXI, complementan el panorama global de las dos anteriores. En línea con libros semejantes publicados en otros idiomas, estos aparecen como pequeñas enciclopedias de consulta, con retratos de las figuras históricas y notas biográficas muy cortas, ninguna de más de dos páginas. Aunque no se ocupan específicamente de ciencia, sí incluyen un buen número de científicos, muchos aún vivos, pertenecientes a diferentes campos de las ciencias y las ingenierías. *Todos somos genios* (Maltés, Torres, 2018) destaca a los ganadores de los premios Nobel, mientras que *Todos somos genios 2* (Maltés, Torres, 2019) a figuras que, si bien sobresalieron en sus campos, no recibieron aquel reconocimiento.

Andrew Maltés y Arturo Torres son los autores de ambos libros. Su propósito es brindar referentes, algunos femeninos, para las nuevas generaciones. Estos referentes comparten la característica de ser genios, personas sin tacha ética o intelectual que destacan entre las demás y solo piensan en el bienestar de la humanidad. Sin duda un estándar muy alto para los lectores. Incluso reaparece el tema de que la vocación por la ciencia surge desde la niñez: los genios son ante todo niños científicos genios, algo que transmiten las ilustraciones, que los representan como adultos aniñados. Por medio del examen de las vidas de esos genios, los lectores se dan cuenta de que todos podemos llegar a ser como ellos. Solo debemos hacer los esfuerzos individuales correspondientes, poner de nuestra parte, aun cuando las notas no dan detalles sobre las motivaciones o los contextos sociales que permitieron alcanzar el objetivo en cada caso, más allá de algunas dificultades generales, especialmente para las mujeres.

Este punto lleva a la última obra que se comentará aquí, también de carácter enciclopédico. Antes, es importante mencionar la dificultad que tuvieron los coordinadores de la Serie Juvenil de Colciencias a finales del siglo XX para hallar mujeres científicas. Aguirre (2004, p.84) lo describe así: “Conseguimos 19 nombres, sin encontrar ni una mujer. Ellas siempre estuvieron detrás de ellos; sólo se destacaban en labores sociales y comunitarias”. Con gran esfuerzo, lograron sumar dos nombres, que conformaron los únicos volúmenes dedicados a mujeres científicas en la colección: *Virginia Gutiérrez de Pineda: observadora silenciosa, maestra apasionada*, de Carlos Andrés Barragán (2001), y *Soledad Acosta de Samper: una historia entre buques y montañas*, de Carolina Alzate (2003).

Dos décadas después, algo importante cambió, pues encontrar mujeres científicas, del pasado y contemporáneas, se convirtió en una labor mucho menos titánica. Aquello que parece haber cambiado, en sintonía con propuestas clásicas de la historia feminista de la ciencia (Schiebinger, 1989), es que la búsqueda dejó de preguntar “¿por qué hay tan pocas mujeres en la ciencia?”, y pasó a preguntar “¿por qué hay tan pocas mujeres en la ciencia de las que estemos al tanto?” Esto implicó que quizá el número de científicas no era realmente bajo, como asumió la Serie Juvenil de Colciencias, sino que se ignoraba o disminuía su presencia por razones sociales, culturales o políticas. Así lo da a entender el libro *Cuentos de buenas noches para niñas rebeldes: 100 mujeres extraordinarias de Colombia y Ecuador*, de Tatiana Andrade, Juliana Muñoz Toro y Lina Toro (2021).

Como sugiere el título, el libro se ocupa de mujeres pertenecientes a muchos campos, incluidos los científicos. Sin afirmarlo directamente, la obra se enmarca en las preocupaciones por incrementar el número de mujeres en la ciencia, que se conectan con acciones como la declaración por las Naciones Unidas del 11 de febrero como el Día Internacional de la Mujer y la Niña en la Ciencia. Darles más visibilidad a las mujeres científicas, desde perspectivas históricas y contemporáneas, se entiende como un modo de promover estas vocaciones en las niñas, mediante referentes femeninos que les ayudarán a integrar la próxima generación de científicas.

El libro apunta en esta dirección, retomando un diseño editorial semejante al de *Todos somos genios:* las ilustraciones también representan a las científicas como mujeres aniñadas, genios cuyo amor por la ciencia se remonta a la niñez y que las niñas lectoras pueden emular, aunque tampoco se den detalles sobre motivaciones y contextos, excepto dificultades generales para destacarse en ambientes ampliamente masculinizados que cualquier niña puede vencer con su fuerza de voluntad. En este sentido, los tres libros siguen la línea de ese feminismo liberal en formato Disney que Macarena García González (2021, p.112-117) encuentra en otros proyectos editoriales de LIJ, donde la genialidad femenina se asocia con la rebeldía individual, el empoderamiento resulta de esfuerzos personales sin base en factores sociales y el éxito implica la participación en el poder dominante, no su transformación.

Este recorrido sugiere que, sea mediante colecciones editoriales u obras individuales, la LIJ colombiana despliega un interés importante por acercar a los lectores a científicos nacionales y extranjeros destacados para la historia del país, particularmente en proyectos como la Expedición Botánica y la Comisión Corográfica, bajo el supuesto de esa mezcla de instrucción moral y entretenimiento racional que Secord identifica en la LIJ del siglo XVIII y que se conecta con la dimensión educativa que describen algunas estudiosas del género. Cabe, así, preguntar quién es, en definitiva, ese científico que reposa en los libros y los manuales de historia, según la LIJ colombiana.

La respuesta es más o menos uniforme, con matices relevantes. Hay cierta obsesión por presentarlo como una unidad bastante estable en el tiempo, que define sus gustos, inclinaciones y potencial en la niñez y permanece inamovible e incorruptible hasta la muerte, a pesar del sinfín de obstáculos que le impone su medio. También hay cierto compromiso con problematizar lo menos posible el imaginario del científico como un sabio o un genio, que sirve de guía epistémica, moral y espiritual a toda la nación, porque sus motivos se condensan en descubrir la verdad de la naturaleza y garantizar el bienestar de la humanidad. Sin embargo, es valioso que las obras exploran otros motivos, asociados con aspectos contextuales como la guerra, la política, la economía o el carácter internacional de la ciencia, además de alguna mención a las mujeres, sin detalles concretos sobre qué significa y qué implica ser una mujer científica.

El interrogante es cómo esta versión del científico histórico contribuye en últimas a fortalecer las vocaciones científicas y, de paso, reducir las brechas de género. Vélez de Piedrahita (2021, p.32) advierte que los textos escritos por niños no deben tomarse como ejemplos de LIJ, pues lo que escribe un niño “no sirve para educar o divertir a otros niños sin enseñar errores”. Más allá de si la valoración es correcta, su planteamiento destaca un aspecto que a veces se pasa por alto y es que no son niños quienes escriben las obras de LIJ, sino adultos. Y son esos adultos quienes, al ocuparse de ciencia, difunden ciertos imaginarios científicos entre sus lectores, lectores que, como se ha insistido, son niños y jóvenes, pero también otros adultos. Esos imaginarios científicos se proponen, entre otras cosas, ampliar el interés por la ciencia, apoyar su respaldo social y así iniciar a los niños y los jóvenes en el diálogo cultural sobre la ciencia en la sociedad.

Nicholas Bullot, William Seeley y Stephen Davies (2017) aseguran que las relaciones entre el arte y la ciencia reflejan históricamente una codependencia. Parte de esta se expresaría en las representaciones artísticas de la ciencia, de sus descubrimientos, teorías y conceptos, de sus personajes, contextos y procesos, entre otros. Estas representaciones llevan a los espectadores a vivir experiencias estéticas que suscitan juicios sobre las propias obras de arte, pero también sobre la ciencia. Más interesante aún, para comprender ciertas formas contemporáneas de subjetividad y cultura, es que dichas experiencias estéticas se levantan sobre las representaciones de la ciencia que el arte pone ante el espectador. La obra de arte de temas científicos (y la obra literaria es un ejemplo de ello) permite desplegar una representación de la ciencia que se apropia de algunos elementos y descarta otros, que irradia optimismo o exhala pesimismo, que puede ser crítica o reverente. El conjunto de estas representaciones es lo que aquí se denomina un imaginario científico.

La obra literaria puede concebirse, siguiendo a Michel Foucault (2005), como uno de los procedimientos de las sociedades contemporáneas para controlar, seleccionar, organizar y redistribuir la producción del discurso científico. La pregunta de fondo es si las obras literarias funcionan como impulsoras o críticas del lugar de la ciencia en la sociedad actual. Los imaginarios científicos tienen dos consecuencias importantes en este aspecto. Primera, instancian diversos compromisos – tácitos o explícitos, de apoyo o rechazo – con ciertos proyectos y poderes políticos, sociales y económicos que rodean a la ciencia. Segunda, sirven de respaldo para que los espectadores emitan juicios, considerados bien fundados gracias a la representación artística, sobre otros imaginarios científicos en la formación de las subjetividades y las culturas de su tiempo. La relación entre experiencia estética e imaginarios científicos se vuelve relevante para comprender cómo la exposición a las obras literarias puede condicionar diversas formas de autorreconocimiento frente a la ciencia.

Surge, así, la inquietud de qué tan conveniente – y quizá qué tan plausible – es representar al científico histórico como un genio y un sabio cuyas inclinaciones científicas se definen ineluctablemente en la niñez. ¿Tienen que verse los lectores así para cruzar el portón de la ciencia? ¿Cómo pueden motivarse aquellos que no encuentran en sí mismos la fortaleza para alcanzar ese pedestal? La dificultad de un juego contribuye a mantener el interés en el juego. Si la ciencia es tan difícil que solo los niños sabios y geniales pueden dedicarse a ella, ¿qué podría inducir al resto de nosotros, conscientes de nuestra inteligencia promedio, nuestras capacidades limitadas y nuestros gustos mundanos y triviales, a gastar tiempo valioso en temas de ciencia? El estándar es tan alto que resultaría más razonable abandonar el juego. Cuando la LIJ colombiana representa a los científicos ficticios, estos suelen mostrarse más cercanos. No se ofrecen como ídolos para admirar, sino opciones de vida que cualquiera puede emular con algún éxito, incluso si se recurre a los estereotipos típicos de la literatura occidental (Escobar Ortiz, en preparación). Sin duda es posible, y quizá culturalmente más conveniente para promover las vocaciones científicas entre niños, niñas y jóvenes, hacer algo análogo con el científico histórico: revelar su valor e importancia sin necesidad de inclinarse ante el monumento en señal de veneración. Por ahora, este acercamiento ha sido minoritario en la LIJ colombiana.

**Tabla 1:** : Obras informativas de temas científicos

Título	Autores	Año
**Textos biográficos**		
*Las siete vidas de Agustín Codazzi*	Beatriz Caballero (texto)	1994
*Codazzi, el señor que dibujaba mapas*	Beatriz Caballero (texto) Olga Cuéllar (ilustraciones)	1994
*Caldas: precursor del patriotismo científico*	Andrés Olivos Lombana	1998
*Galileo Galilei, y sin embargo se mueve*	Germán Puerta-Restrepo	2005
*¡Soy Caldas! Biografía de Francisco José de Caldas*	Stefan Pohl Valero (texto) Olga Cuéllar (ilustraciones)	2009
*Maquiavelo: ¿un científico moderno de la política?*	Enrique Ferrer Corredor	2018
*Todos somos genios*	Andrew Maltés y Arturo Torres (textos) Omar Andrés Penagos (ilustraciones)	2018
*Todos somos genios 2*	Andrew Maltés y Arturo Torres (textos) Omar Andrés Penagos (ilustraciones)	2019
*Cuentos de buenas noches para niñas rebeldes: 100 mujeres extraordinarias de Colombia y Ecuador*	Tatiana Andrade, Juliana Muñoz Toro y Lina Toro (texto) Varias ilustradoras (ilustraciones)	2021
**Reconstrucción de procesos históricos**		
*La Expedición Botánica contada a los niños*	Elisa Mújica (textos) Ángela Medellín (ilustraciones)	1978
*La aventura de la Expedición Botánica*	Beatriz Caballero (textos) María Cristina Londoño, Jorge Langlais y Birgit Schlegel (ilustraciones)	1983
*Mutis y la Expedición Botánica*	Gonzalo España (texto)	1999

Fuente: Escobar Ortiz (2023).

**Tabla 2:** : Obras literarias de temas científicos

Título	Autores	Año
**Biografía novelada/novela biográfica**		
“José Celestino y el dragón” (cuento) en *Pasado y porvenir de la Expedición Botánica*	Elisa Mújica (texto)	1985
“Aventuras del príncipe Celestino” (cuento) en *Cuentos para niños de La Candelaria*	Elisa Mújica (texto) Ángela Medellín (ilustraciones)	1993
*El río de la vida. Manuel Elkin Patarroyo y su lucha contra la malaria*	Jairo Aníbal Niño (texto)	1994
Caldas. El inventor de lunas*	Jairo Aníbal Niño (texto) Silvia Gómez (ilustraciones)	1995
Humboldt. El muchacho de la cruz del sur*	Gonzalo España (texto) Silvia Gómez (ilustraciones)	1996
Claude Véricel. El amigo de los animales*	Celso Román (texto) Silvia Gómez (ilustraciones)	1997
Francisco Javier Cisneros. El que comunicó con rieles las comarcas*	Pilar Lozano (texto) Cristina Salazar (ilustraciones)	1997
José María Villa. El violinista de los puentes colgantes*	Pilar Lozano (texto) Cristina Salazar (ilustraciones)	1997
Codazzi. El siete leguas*	Beatriz Caballero (texto) Silvia Gómez (ilustraciones)	1997
Lino de Pombo. El sabio de las siete esferas*	Germán Espinosa (texto) Hernando Carrizosa (ilustraciones)	1998
José Celestino Mutis. El sabio de la vacuna*	Gonzalo España (texto) Silvia Gómez (ilustraciones)	1998
Alejandro López. A la medida de lo imposible*	Irene Vasco (texto) Elsa Zambrano (ilustraciones)	1998
Julio Garavito. De Colombia a la Luna*	Sandro Romero (texto) Claudia García (ilustraciones)	1998
Ezequiel Uricoechea. El niño que quería saberlo todo*	Celso Román (texto) Claudia García (ilustraciones)	1998
Federico Lleras Acosta. La guerra contra lo invisible*	Germán Espinosa (texto) Cristina Salazar (ilustraciones)	1998
Jean Baptiste Boussingault. El padre de la agricultura moderna*	Gonzalo España (texto) Liliana Ramos (ilustraciones)	1998
Pierre Bouguer. El maestro del sabio*	Gonzalo España (texto) Hernando Carrizosa (ilustraciones)	1998
Manuel Uribe Ángel. El médico y geógrafo que amó a su país*	Pilar Lozano (texto) Liliana Ramos (ilustraciones)	1998
José Jerónimo Triana. El caballero de las flores*	Santiago Díaz Piedrahita (texto) Silvia Gómez (ilustraciones)	1999
Virginia Gutiérrez de Pineda. Observadora silenciosa, maestra apasionada*	Carlos Andrés Barragán (texto) Cristina Salazar y Marta Calderón (ilustraciones)	2001
Soledad Acosta de Samper. Una historia entre buques y montañas*	Carolina Alzate (texto) Juanita Isaza (ilustraciones)	2003
Agustín Codazzi: el caballero andante**	Carlos Nicolás Hernández Camacho	2010
Mutis y la minería. Amorosa mutisia**	Celso Román	2010
*Francisco José de Caldas: un camino a las estrellas*	Álvaro Vélez (truchafrita) Gabriel Jaime Gómez Carder	2019
**Novela histórica**		
*Los pies en la tierra, los ojos en el cielo*	Gonzalo España (texto) Iván Chacón (ilustraciones)	2008
*La última correría del sabio Mutis*	Gonzalo España	2017

*Serie Juvenil de Colciencias

**Biblioteca Colombiana Ilustrada

Fuente: Escobar Ortiz (2023).

## Data Availability

No está en repositorio.

## NOTA

1 En esta y otras citas de textos en otros idiomas, la traducción es libre.
